# Expanding Education Researchers’ Access to Classroom Observation Data With a Remote and Cost-Effective Video Data Collection Protocol

**DOI:** 10.1007/s11121-024-01659-w

**Published:** 2024-03-22

**Authors:** Leigh McLean, Paul Espinoza, Kati Tilley, Lori Foote, Nathan Jones, Ben Kelcey

**Affiliations:** 1 Center for Research in Educational and Social Policy, University of Delaware, 125 Academy Street, Newark, DE 19716 USA; 2 T. Denny Sanford School of Social and Family Dynamics, Arizona State University, 951 Cady Mall, Tempe, AZ 85287 USA; 3 College of Education, Criminal Justice, and Human Services, University of Cincinnati, 2610 University Circle, Cincinnati, OH 45221 USA; 4 College of Education and Human Development, Boston University, 2 Silber Way, Boston, MA 02215 USA

**Keywords:** Classroom observation, Classroom research, Research methods, COVID-19

## Abstract

The onset of the COVID-19 pandemic and associated long-term shifts to virtual instruction among most US schools presented notable challenges among education researchers. Ongoing projects conducted in school settings experienced sudden losses of access to teacher and student participants, in many cases leading to severe interruptions to data collection efforts. Perhaps most notably, upon returns to in-person instruction in the 2021/22 academic year most schools instigated strict policies limiting the number of non-school personnel who could enter school buildings, including researchers conducting in-person data collections. As such, many researchers had to find alternative means to gather data. In this paper, we offer a new protocol that we created in response to these challenges that allows for the secure and fully remote collection of video data in school settings. This new protocol not only addressed the immediate needs of the focal study but also addresses some of the most notable barriers to collecting classroom video data in the field of education research at large. In this paper, we describe the initial development and application of this protocol among a local study of elementary teachers, as well as the scaling of this protocol in a study of elementary teachers in multiple states. It is our hope that this protocol can expand education researchers’, practitioners’, and policymakers’ access to classroom video data.

**Supplementary Information:**

The online version contains supplementary material available at 10.1007/s11121-024-01659-w.

The onset of the COVID-19 pandemic in March 2020 and associated long-term shifts to virtual instruction among most US schools ushered in an unprecedented set of challenges to the US education system. Among education researchers, ongoing projects conducted in school settings experienced sudden losses of access to teacher and student participants, in many cases leading to severe interruptions to data collection efforts. While most US schools returned to in-person instruction in the 2021/22 academic year, these returns did not fully revert all barriers to conducting school-based research. The structure and routines of classrooms changed fundamentally with the inclusion of safety barriers and masking requirements, and most schools instigated strict policies limiting the number of non-school personnel who could enter school buildings, including researchers conducting in-person data collections. As such, many researchers had to find alternative means to gather data.

During the COVID-19 pandemic, authors of this paper were conducting a large, school-based study exploring how teachers’ and students’ emotions (i.e., enjoyment, anxiety) contribute to classroom processes. This study began in the Fall of 2018 and data collection was originally planned to occur among three unique cohorts of elementary teachers and their students: one cohort in the 2018/19 year, one in the 2019/2020 year, and one in the 2020/21 year. Planned in-person data collection activities included paper-and-pencil student surveys administered by research team members, as well as extensive classroom video observations conducted by research team members using a project video recording equipment. Data collection for this project was paused in the 2020/21 year due to prolonged school closures and the study timeline was extended to allow for two additional cohorts to undergo data collection in 2021/22 and 2022/23. However, upon beginning data collection among the 2021/22 cohort, the research team was no longer able to conduct in-person data collections. Shifts from paper-and-pencil student surveys to electronic surveys were fairly straightforward, but capturing classroom video observations remotely presented the team with a unique challenge. As any observation researcher would likely attest, gathering video data in school settings where privacy and security restrictions abound and where observation work can be intrusive to the teacher is challenging under typical circumstances. Adding to this, the need to conduct observations completely remotely and the astronomical increases in stress experienced by teachers due to the pandemic (Diliberti et al., 2021; Kaufman & Diliberti, 2021; Pressley, 2021), and it was clear that we needed to be thoughtful and resourceful in our solutions. Fortunately, the research team was able to develop a new protocol that allowed for the secure and fully remote collection of video data in school settings. This new protocol not only addressed the immediate needs of the focal study but also addresses some of the most notable barriers to collecting classroom video data in education research at large. The research team saw so much success with this protocol among the 2021/22 cohort that we opted to continue collecting video data remotely among the following cohort in 2022/23 and have since piloted this approach in a national, multi-site study conducted by the PI. In addition to allowing us to continue collecting classroom observation data during a time where in-person classroom access was limited, we feel this protocol could be used as an alternative to in-person video data collection in education research moving forward. Here, we present this protocol in the hopes that researchers, practitioners, and policymakers can benefit from increased access to classroom video data.

## Utility and Challenges of Classroom Observation Data

Classroom observations provide education researchers with firsthand, in-depth views of important classroom processes that are free from some of the most common types of bias in human-subjects research (Kane & Staiger, 2012). Classroom observations based on video data are especially useful as they offer unparalleled opportunities for the systematic training of observers and for the more robust (re)establishment of inter-rater reliability that is more challenging to achieve with live observations (Casabianca et al., 2013; Haidet et al., 2009). Classroom video data are also high yield, as the same videos can be assessed using multiple tools to address a variety of research questions (McLean & Connor, 2018), and are a cornerstone of mixed methods research (Lindorff & Sammons, 2018) as they can be assessed using both qualitative and quantitative approaches and can be analyzed in combination with other data to inform complex research questions.

In contrast to their utility, classroom video data are challenging to collect and store. For researchers, collecting these data can often be costly given the equipment and person-hours necessary, especially considering that best practices necessitate the collection of multiple observations from each classroom to achieve an accurate view of classroom processes. For participants, scheduling a time for these observations can be difficult given teachers’ busy and often changing schedules (Bettini et al., 2017; Walker et al., 2019), and video data collection procedures that involve an outside party coming into the classroom can be stressful and intrusive (Lasagabaster & Sierra, 2011). These factors might ultimately discourage teachers from electing to participate in studies involving classroom video observations. Lastly, classroom video data are typically classified as “highly sensitive data” and as such researchers are limited in the types of equipment and services they can apply to collect and store these data.

Due to these challenges, it is often unfeasible for education researchers to incorporate video data into empirical studies in ways that can be generalized to larger populations. This is unfortunate because education policymakers and stakeholders rely on the results of large-scale, quantitative research to inform policy decisions that have direct implications for teachers and students. For example, the National Assessment of Educational Progress (NAEP) which provides regular reports of US students’ performance in core content areas (NAEP, 2022a) is frequently used to justify the allocation of resources to support teachers and students (NAEP, 2022b). However, due to the inherent challenge of collecting and storing video data in education, observation data are not often incorporated into such research efforts in robust ways. As a result, decisions by policymakers and stakeholders are likely under informed by empirical findings that consider firsthand observations of classroom processes that could serve to inform more precisely the reasons behind patterns detected in quantitative data. Thus, by expanding education researchers’ capacities to collect and store video observation data on a large scale, we can increase the likelihood that policy decisions are made based on research that includes consideration of observed classroom processes.

Another notable barrier is the limited open sharing of resources among education researchers to support the widespread knowledge of best practices in collecting classroom video data. It is not currently the norm for established observation researchers to formally document and make available their video data collection and storage protocols. As well, rapidly changing technologies have shifted the landscape in terms of what possible approaches can be taken to observing classrooms (i.e., new remote technologies that have not yet been applied to education research). Some formal resources do exist, for example, the Best Foot Forward Video Observation Toolkit (Kane et al., 2015), but these are typically geared towards the use of video observations for evaluation and professional learning purposes rather than for research and so do not attend as closely to research-specific issues such as consent processes, videography for the purpose of later analysis, or data security protocols. As a result, many researchers, especially early career researchers and those venturing to collect classroom video data for the first time, must build their own systems from scratch with limited information on what works, what does not, and what tools and technologies are available to them. The widespread sharing of resources that include consideration of more recent technologies and that are streamlined for researchers (i.e., include information about data security, consent processes, etc.) would help ensure that these data are increasingly collected using best practices.

With this paper, our objectives are twofold: first, we want to share a new approach to the collection of classroom video data that addresses some of the most notable barriers to the collection of classroom video data and which we believe can expand education researchers’ capacities to incorporate these data into their research. Second, we hope to take an important step toward the open sharing of information and resources to support best practices in the collection and use of classroom video data for education research. These objectives can contribute to the broader goal of ensuring that education policy decisions are increasingly made based on research findings that incorporate consideration of observed classroom processes.

## Current Approaches to Collecting Classroom Video Data

In Table 1, we provide a summary of all generally accessible (i.e., researchers can feasibly identify, purchase, and use all necessary equipment) approaches to collecting classroom video data known to this investigative team in terms of each approaches’ modality, locations the approach can be applied in, resulting video/audio quality, cost, burden, and security. In addition, we also provide an expanded discussion of the affordances and barriers of each approach.

**Table 1:** Summary of barriers and affordances for each data collection approach

**Approach**	**Modality**	**Location**	**Video/audio quality**	**Cost**	**Burden on researcher**	**Burden on participant**	**Security**
Past approaches							
Videographer in classroom	In-person	Local	High	High	High	High	High
Swivl cameras managed by researcher	In-person	Local	High	High	High	High	High
Swivl cameras managed by participant	Remote	Multi-site	High	Moderate	Moderate	High	High
Virtual meeting recording (e.g., Zoom)	Remote	Local or multi-site	Low	Low	Low	Low	Low
Teacher self-records on own device	Remote	Local or multi-site	Highly variable	Low	Low	Moderate	Low
New approach							
Google Nest cameras + hotspot delivered by researcher	Remote	Local	High	Low	Moderate	Low	High
Google Nest cameras + hotspot shipped to participant	Remote	Multi-site	High	Moderate	Moderate	Low	High

Perhaps the most common approach to collecting classroom video data has been for researchers to conduct in-person video recordings. Typically, recording sessions are scheduled in advance with the teacher and on the day of recording one or more project personnel come to the classroom and record, often moving throughout the classroom during the lesson. This approach usually yields high-quality video data and is relatively free from user error as project personnel are trained in the use of recording equipment and are familiar with the content they need to capture. In this approach, the videographer can also tailor the recording to the needs of the researcher by intentionally capturing certain classroom processes as they occur, for example by zooming in on the materials being used or capturing a closer view of a teacher working with a small group of students. However, this approach is more invasive for the teacher and students and can be overly rigid for teachers who often need to make last-minute changes to their schedules and are frequently interrupted (Kraft & Monti-Nussbaum, 2021). It is also costly as videographers must be paid for their time and travel and was less feasible in the wake of COVID-19 which ushered in stricter policies for outside parties gaining access to schools.

To address these concerns, researchers have more recently turned to remote approaches. We note three accessible remote approaches: First, teachers can capture their own videos using remote recording equipment provided by the researcher. One commonly used example is the Swivl camera, which is placed in a central location in the classroom and connects to a beacon and microphone worn by the teacher. The camera pivots from left to right to follow the teacher’s beacon and records the audio/video surrounding the teacher. While this approach typically yields high-quality audio and video, equipment is expensive for the researcher at between $600 and $900 per camera and can be cumbersome for the teacher in terms of setup and maintenance, and the need to incorporate wearable devices. The use of Swivl cameras can be managed in-person by the researcher (the researcher brings a Swivl camera and sets it up for the teacher), or cameras can be delivered to teachers with the expectation that they set up the system themselves.

A less intensive remote approach is to use the meeting recording function in a video meeting platform (i.e., Zoom). This is much less expensive and more user-friendly; however, it yields lower-quality audio and video and the extent to which the camera can capture an adequate view of the classroom is dependent on the placement of the computer being used in the classroom. This approach is also vulnerable to data security issues, as users must rely on the school’s and researchers’ internet connections which may be unreliable and/or open to outside interference. Some past efforts have also asked teachers to record classroom sessions using their own handheld device (e.g., smartphone, tablet), and while this is likely the least expensive option for the researcher, it is unlikely to result in consistently high-quality audio and video across teachers and devices and is the most prone to user-error. This approach also has security issues as teachers must send completed video files to researchers and there are limited ways to do this securely that are not overly cumbersome to the teacher. As well, there is no way for the researcher to guarantee that teachers permanently delete these videos from their personal device.

## A New Approach

Based on the limitations above, it is clear that there is a need for a new approach that (1) is fully remote; (2) reliably captures high-quality audio and video; (3) is user-friendly for both the researcher and participant; (4) is minimally invasive; (5) is flexible for teachers to fit into their constantly shifting schedules; (6) is cost-effective; (7) is secure and reliable; and (8) can be implemented on a large scale and across multiple locations. With these needs in mind, we piloted (in a local study) and later scaled (in a multi-state study) a novel approach to remote classroom video data collection wherein self-recording equipment is delivered to teachers who then capture their own observations over a period of 1 to 2 weeks.

We do want to note one large-scale, multi-site video data collection effort that applied an approach with overlapping features: from 2010 to 2013, the Measures of Effective Teaching Project (MET, Kane et al., 2013, 2014) used customized panoramic digital cameras delivered to and operated by teachers to capture video data among US classrooms. While this protocol was implemented with success, it is limited in its ability to be replicated by other research teams given that the equipment used was developed exclusively for the project and as such are less accessible to those hoping to use a similar approach. It is also unclear whether this approach would meet today’s institutional standards for data security, and it does not incorporate the most recent remote video recording technology. The new protocol described here offers more recent and accessible technologies that can be used with ease by both the researcher and participant, as well as a high level of security.

### Equipment

Observation kits are delivered to teachers (either personally by project staff in a local study or via mail in a multi-state study) that contain a remote camera, a secure Internet hotspot, and detailed directions for setup, use, and troubleshooting. The cameras are home security cameras, which to our knowledge have not yet been widely used for the purposes of education research. Home security video quality has improved substantially over the past decade, and almost all home security systems are now managed through private online portals controlled by the user (in this case, the researcher). After a review of all viable home security providers, we determined that the equipment and user platform provided by Google best met the above needs. The Google Home system provides users with an online management platform called Google Nest where they can view and download security camera footage, with various subscription options for video storage capacity and length. Users can purchase any one of Google’s home surveillance cameras to be used in conjunction with the Google Nest platform, with both indoor and outdoor, and wired and wireless cameras available. We determined that Google’s Nest Indoor Wired camera most closely met our needs. The Indoor Wired camera measures just under 4 inches tall and 2.24 inches wide, and weighs approximately 14 oz. The camera face is permanently affixed to a small, weighted circular base that can be placed on any flat surface facing the focal area which the user wants to capture. This camera offers clear video and audio quality and captures a wide-angle (approximately 135 degrees) view of whichever area it is facing, making it possible to capture most of the instruction occurring within a classroom at any given time, especially if the camera is placed on a surface 4 to 6 feet high such as a desk or a bookshelf. As well, we opted for wired cameras so that participants would not need to worry about camera battery life while recording. Google also offers outdoor battery-powered cameras without wires, which can be mounted on walls. Google Nest Indoor and Outdoor cameras are outfitted with a 2-megapixel color sensor, 6× zoom-in capability, and a 16:9 aspect ratio. Video can be recorded in up to 1080p at 30 frames per second, with high-definition options. Audio is recorded with a full-duplex 2-way audio that includes noise cancellation, resulting in clear audio that picks up multiple voices well.

The Google Nest Indoor Wired camera relies on an Internet connection to provide a live video feed to the user’s Google Nest account, with recording automatically initiated when the camera is plugged in. All video footage captured by the camera is stored for 3 days on the user’s Google Nest account, with options for longer-term storage via subscription available. Due to concerns about the reliability and security of school Internet connections, we opted to purchase our own secure Internet hotspots to be used in conjunction with the cameras. After an initial review of hotspot devices and, later, a review of the Internet coverage quality in each of the states with participants in the multi-state study, we opted to purchase Verizon Jetpack hotspots and associated Verizon data packages; however, this protocol should work with most hotspot models and Internet service providers. These devices can be set up by the researcher to automatically pair when later turned on by participants during a recording session.

### Conducting an Observation

Before a teacher records their first session, all teacher and student consent procedures are completed, and teachers are asked to familiarize themselves with the recording equipment and instructions. When ready to record, the teacher first attends to the removal of unconsented students either from the view of the camera or by removing them from the classroom altogether. A teacher can set up the camera at a mid-point in the classroom and move unconsented students behind the camera, out of view. Once students are appropriately placed in the classroom, the teacher follows researcher-provided instructions for turning on the camera and hotspot, ensuring the two devices have successfully paired, and placing the camera. Once turned on and paired with the hotspot, the camera will automatically start recording audio and video, and the teacher then conducts their lesson. Once the lesson is complete, they unplug both devices and store them for later use. Unplugging the camera automatically stops the recording, and the video footage is immediately available to the researcher via the Google Nest platform. If at any time during a recording the connection between the camera and hotspot is interrupted, or if the camera is accidentally unplugged, the teacher can plug in and pair the devices again and recording will resume. When the teacher has completed all recording activities, the researcher can either retrieve the kit in person (if local), or the teacher can use pre-paid postage provided to ship the camera kit back. If at any point an unconsented student accidentally appears on camera, the researcher can trim or blur portions of the video as necessary before transferring the video to long-term storage.

If necessary, the researcher and teacher can communicate about when a recording will take place, and the researcher can access the live feed of a recording via their Google Nest account to give feedback on camera placement, removing unconsented students, etc. However, we have found that teachers are generally able to successfully place cameras on their own following our instructions, and that the wide-angle view captured by the cameras yields viable video data even when camera placement is not ideal.

### Downloading and Storing Video Data

The researcher can access completed recordings via their Google Nest account, and from there can download, edit, and transfer all videos captured to longer-term storage devices. The researcher can opt to receive a notification via their Google Nest account each time a recording is completed by a teacher. Alternatively, if the researcher has subscribed to longer-term video data storage, they can simply log into their account regularly to download any videos that were completed by teachers since their last login and transfer them to a secure server or external storage device for long-term storage. These data access, download, transfer, and storage procedures were approved by the PI’s IRB board as well as by the institution’s Office of Educational Technology who completed an additional investigation regarding the security of the Google Home system. Thus, we are confident these procedures would meet the data security standards of most research institutions.

### Cost Comparison

We conducted a detailed analysis illustrating the differences in cost between this new approach and the most traditional approach of in-person video recording by researchers. Table 2 provides the following costs associated with this new approach: the price of all equipment, subscription fees for a month of subscriptions to support data collection, and the average price of shipping one kit to and from another state. Prices are then explicated to provide an estimate of what it would cost to use this approach to collect both in-person and out-of-state (estimated separately) observations from 30 teachers over a 2-month period using five observation kits. In Table 3, we provide a comparative analysis for an in-person video observation protocol collecting the same amount of data in the same time span. Specifically, we estimate the cost of all associated equipment (not including a computer), the hourly wages for a non-benefits-eligible hourly worker to conduct the observation, and the mileage reimbursement estimated for one round trip to and from a school. We then use these to provide an estimate for the cost of collecting in-person observations from 30 teachers over a 2-month period using five observation kits.

**Table 2:** Cost analysis of the proposed remote video data collection protocols among both local and out-of-state participants

**Item**	**Price per unit**	**Price for 30 observations over 2 months using 5 kits**
Equipment		
Tablet	$1000	N/A
Google Nest Indoor Camera	$100	$500
Verizon Jetpack Hotspot	$150	$750
Carrying case	$15	$60
Equipment total	$1,265	$2,310
Subscriptions		
Google Home w/ 10-day storage	$6	$12
Verizon unlimited data plan	$40	$400
Subscriptions total	$106	$412
Total (local study)		$2,722
Shipping (multi-site only)		
Shipping materials	$4	$120
Shipping to participant	$25	$750
Pre-paid return shipping label	$27	$810
Shipping total	$56	$1,680
Total (multi-site study)		$4,402

**Table 3:** Cost analysis of a comparable in-person video data collection protocol among local participants

**Item**	**Price per unit**	**Price for 30 observations over 2 months using 5 kits**
Equipment		
Handheld camera	$230	$1,150
Tripod	$25	$125
SD card with adequate storage	$45	$225
Carrying Case	$15	$60
Equipment total	$315	$1,560
Personnel		
Hourly Wages (6 h @ $18)	$108	$3,240
Mileage (15-mile round trip)	$10	$300
Personnel total	$118	$3,540
Total (local study)		$5,100

Based on this analysis, it is clear that the proposed protocol is less expensive: applying this remote protocol to local participants is just 53% of the cost of the comparable in-person protocol, with this dropping to about 35% of the cost if the researcher already has a device on which to manage their Google Nest account. Applying the remote protocol to participants in multiple states is 87% of the cost of the comparable in-person protocol, again with this dropping to about 65% of the cost if the researcher already has a device on which to manage their Google Nest account. Costs associated with other approaches (Swivl cameras, etc.) were not estimated in detail for this analysis; in general, the cost of using Swivl cameras falls between these two approaches, and the costs of using virtual meeting platforms or asking teachers to use their own devices were the least expensive (however, these last two approaches come with notable data quality and security issues, see Table 1).

### Most Notable Strengths

It has traditionally been recommended that research personnel be present during video observations to set up equipment and conduct the recording (Haidet et al., 2009). This new approach circumvents this need, as participants can set up and operate all associated equipment with ease, and high-quality video and audio can be captured even in cases where setup has been less than ideal. As such, a first strength that we note is that this approach is more feasible and cost-effective compared to sending in-person videographers to classrooms and/or using more expensive and complex remote recording equipment. Researchers can use this protocol to incorporate classroom video observations into proposals without overstraining their budgets, eventually leading to more funded empirical efforts that include classroom video data.

Second, the increased flexibility and decreased stress introduced by this approach compared to in-person observations can increase the number of teachers who can participate in studies that include observations. This new protocol gives teachers full access to all recording equipment over a 1- to 2-week recording period during which they can complete recordings at their convenience, rather than needing to stick to a previously scheduled time that may or may not work for them the day of. As well, the fact that no outside personnel are entering the classroom can reduce stress, minimize interruptions, and more generally reduce participant reactivity to data collection (Paterson, 1994). We offer that this approach may be especially helpful in collecting video observations among special education teachers/classrooms, as these teachers are likely interrupted at higher rates. As such, this protocol could be a viable way to increase the number of studies applying observation methods to special education classrooms, as these contexts are currently under-informed by observation methods (Jones & Brownell, 2014). Another source of flexibility afforded by this protocol is the ability to either conduct asynchronous observations as was done by this group where the teacher records themselves and the video is assessed at a later time, or to conduct in-the-moment remote observations using the live stream feature on the researchers’ Google Nest account.

Third, if adopted by leaders of large-scale, multi-site studies this approach could expand the fields’ capability to incorporate classroom video data into quantitative empirical efforts that can be generalized to all teachers in a region/country/etc. Given the costliness associated with large-scale, national efforts to collect video data, data sets that include classroom video data collected from multiple states are rare. Researchers leading large studies who are interested in incorporating observations may need to restrict their collection of video data to smaller subsamples, resort to cheaper but lower-quality approaches, or forego observations altogether. By applying this approach, education researchers can more easily incorporate high-quality video observations into their multi-site studies with the budgets available from funders.

### Limitations and Recommendations

We also want to note some limitations of this approach and provide recommendations for overcoming these. First, while resulting video and audio quality are more than adequate for the application of classroom-level observation tools, we are still unsure if this approach could yield observation data that can support the application of very detailed, student-level observation tools or tools that assess close interactions. We have not tested the zoom functions of the Google Nest Indoor camera during live observations to ensure adequate video and audio for the coding of closer scenarios such as a small group of students working with the teacher or the teacher working with an individual student, or individual student/teacher or student/student interactions. We are planning to assess this in future work; however, researchers seeking to use this protocol to assess closer views of individual students should test this carefully first before planning a full implementation of the protocol. Users could also pair elements of this protocol with elements of other protocols and/or other equipment (i.e., equipping teachers with microphones, using different security cameras with additional functions) to capture the necessary detail in their videos.

Second, we want to note that Google’s home security systems including associated equipment and hosting platforms (i.e., Google Nest, Google Home) are continually undergoing updates which can change the ways they operate within this protocol. For example, during the use of this protocol in the focal studies, Google restricted the viewing and accessing of video data gathered using our camera model to the Google Nest app, necessitating that we purchase a tablet to facilitate our downloading of observations instead of managing the protocol from a desktop computer as we had been. This is an example of how quick shifts in technology and associated user interfaces can impact how researchers use and interact with this protocol, and we broadly caution that the constantly evolving nature of technology might result in others implementing this protocol to need to adapt it accordingly in the future.

Third, depending on the volume of video data to be collected and the Google Nest subscription level chosen by the researcher, it could be project staff need to dedicate substantial amounts of time to downloading, editing, and storing video data. If a researcher opts not to subscribe for longer-term storage in the Google Home app, they will need to access and transfer videos as they become available or risk losing those videos permanently. Due to this, we strongly recommend that researchers applying this approach opt for a Google Nest subscription that affords them longer-term video storage.

### Preliminary Evidence of Usability and Feasibility

While developing this protocol in the summer prior to using it in the first (local) study, the research team tested all equipment and procedures in a lab setting with the goal of maximizing the protocol’s feasibility and usability. Adjustments were made iteratively as needed until the research team felt they had achieved a near-final set of materials and instructions. In a final trial of the protocol’s usability and feasibility, a university colleague at the project’s awarded institution who was not familiar with the protocol or equipment followed the protocol independently to capture video footage of an empty university classroom. This individual was able to successfully capture viable (i.e., a clear view of most of the classroom, discernable audio throughout the classroom) video data without the assistance of the research team and reported no difficulties in following the instructions or using kit equipment. As well, the research team was able to immediately access, download, and edit the video captured via the associated Google Nest account. We also asked this person to record the time it took for them to set up all equipment, and they were able to do so in under 5 min. This trial led us to determine with confidence that the protocol was usable and feasible for local study teachers to apply in an elementary classroom setting in the upcoming academic year.

Throughout each video data collection phase in the local study, the research team offered phone and/or virtual meetings for any teachers who wanted to review and/or test the protocol prior to conducting their own observations, with offers of these services included in scheduling emails as well as noted in the protocol instructions included with the kits. We also offered to check camera views in real-time upon the start of a teachers’ observation in order to verify successful camera placement. Despite the availability of these supports, the vast majority of teachers in the local study opted to apply this protocol completely independently of research team assistance. Throughout the following data collection phases, 93% of teacher participants in the local study were able to successfully provide video data to the research team. As well, all missing video data were due to reasons unrelated to the protocol (i.e., teacher scheduling issues, extended absences, etc.), and no instances of equipment failure or protocol-related barriers to capturing videos were reported. All videos gathered using this protocol were determined to be viable and were later assessed in the lab successfully by study team members. The local study included 35 elementary teachers who ranged in years of teaching experience from 0 to 38 years (mean = 10.4 years, SD 8.34 years), and who were majority female (88%) and White (71%).

In the multi-state study, the protocol remained the same except for the delivery and return methods for the camera kits. In this study, camera kits were shipped to participants’ schools via the United Postal Service (UPS) and participants were asked to personally drop camera kits off at a UPS shipping location upon completion of observations. The research team provided pre-paid return shipping labels, and kits were shipped back. This adapted protocol was first piloted among a small number of participants in the multi-state study, and in this pilot, the research team shipped camera kits to participants in four US states that were not adjacent to the research team’s state. Throughout the piloting process, the research team remained in email and phone contact with participants and solicited feedback on their ability to carry out the entire adapted protocol. All pilot participants successfully received their kits, conducted video observations, and returned kits to the research team. No pilot participants reported any notable difficulties with any aspect of the adapted protocol. All videos collected were viable, and no equipment was lost or damaged, though we do note the potential for equipment loss and/or damage should this approach be applied among large numbers of teachers nationally or internationally, and recommend researchers allow for these losses in their budgets. The multi-state pilot study included 10 first-year teachers who were all female and White.

We did not collect any formal data from teachers regarding their perceptions of the usability and feasibility of this protocol and use of the associated equipment, and so we present the above as preliminary evidence of this protocol’s usability among teachers in both local and multi-state research studies. We look forward to expanding on this preliminary evidence via and more formal evaluations of the implementation of this protocol in future studies that include direct user input.

## Benefits to the Field

In addition to providing an alternative route to video data collection in school settings under the unique and more restrictive circumstances of the 2021/22 academic year, we see potential for this protocol to expand education researchers’ access to classroom video data on a large scale. By making the collection of classroom video data more feasible, cost-effective, and scalable, more studies (and larger, multi-site studies) can incorporate these types of data. Lowered burden on the part of the teacher would likely increase teachers’ willingness to participate in observation studies, and lower costs associated with collecting these data would increase the ability of those seeking funding to incorporate the collection of these data without overstraining project budgets. All of these features could enrich empirical efforts attempting to describe and intervene upon critical teaching and learning processes, with results better able to inform education policy decisions that impact the success of districts, schools, teachers, and students. In addition to benefiting education research and policy, this protocol could also be incorporated into systems for teacher evaluation and professional learning. Classroom observations are widely used in the evaluation of teachers (Cohen & Goldhaber, 2016; Hill & Grossman, 2013) and as tools to facilitate learning and reflection in teacher professional development (Marsh & Mitchell, 2014; Postholm, 2012), and so practitioners in these areas could apply this protocol as well. In sum, we are confident that this protocol can increase the capacity of education researchers and practitioners to incorporate video observation data into their projects and programs to advance the field in meaningful ways.

## Supports for Implementation

We are committed to assisting others in implementing this protocol via the open sharing of relevant materials. In service of this, we have provided the following as Supplementary Documents to accompany this paper: instructions for the setup and use of this protocol by participants, example teacher and guardian consent documents, and text to aid in the creation of IRB protocols, technology reviews, and manuscripts detailing associated procedures. As well, the research team is happy to offer individual consultations to researchers seeking to learn more about this protocol. Any individual interested in individual consultation can reach out to the corresponding author via the email address provided.

## Supplementary Information

Below is the link to the electronic supplementary material.

Supplementary file1 (DOCX 18 KB)

Supplementary file2 (DOCX 15 KB)

Supplementary file3 (DOCX 33 KB)

Supplementary file4 (DOCX 20 KB)

## Data Availability

Data from the focal studies in which this work was performed will be transferred to an online data repository approximately one year after the culmination of each project, which we estimate will be June 2025 for the local study and June 2027 for the multi-state study. Any parties wishing to access data before they are made publicly available should contact the first author using the information provided.

## References

[CR1] Bettini, E., Jones, N., Brownell, M., Conroy, M., Park, Y., Leite, W., . . . Benedict, A. (2017). Workload manageability among novice special and general educators: Relationships with emotional exhaustion and career intentions. *Remedial and Special Education,**38*(4), 246–256.

[CR2] Casabianca, J. M., McCaffrey, D. F., Gitomer, D. H., Bell, C. A., Hamre, B. K., & Pianta, R. C. (2013). Effect of observation mode on measures of secondary mathematics teaching. *Educational and Psychological Measurement,**73*(5), 757–783.

[CR3] Cohen, J., & Goldhaber, D. (2016). Building a more complete understanding of teacher evaluation using classroom observations. *Educational Researcher,**45*(6), 378–387.

[CR4] Diliberti, M., Schwartz, H. L., & Grant, D. M. (2021). *Stress topped the reasons why public school teachers quit, even before COVID-19*. Rand.

[CR5] Haidet, K. K., Tate, J., Divirgilio-Thomas, D., Kolanowski, A., & Happ, M. B. (2009). Methods to improve reliability of video-recorded behavioral data. *Research in Nursing & Health,**32*(4), 465–474. 19434651 10.1002/nur.20334 PMC2713814

[CR6] Hill, H., & Grossman, P. (2013). Learning from teacher observations: Challenges and opportunities posed by new teacher evaluation systems. *Harvard Educational Review,**83*(2), 371–384.

[CR7] Jones, N. D., & Brownell, M. T. (2014). Examining the use of classroom observations in the evaluation of special education teachers. *Assessment for Effective Intervention,**39*(2), 112–124.

[CR8] Kane, T., Kerr, K., & Pianta, R. (2014). *Designing teacher evaluation systems: New guidance from the measures of effective teaching project*. John Wiley & Sons.

[CR9] Kane, T. J., Gehlbach, H., Greenberg, M., Quinn, D., & Thal, D. (2015). *The best foot forward project: A toolkit for video in the classroom how classroom-based, teacher-controlled video can improve the observation process*. Retrieved May 27, 2023, from https://www.gse.harvard.edu/news/uk/15/10/toolkit-video-classroom

[CR10] Kane, T. J., McCaffrey, D. F., Miller, T., & Staiger, D. O. (2013). *Have we identified effective teachers? Validating measures of effective teaching using random assignment. Research paper. MET project*. Bill & Melinda Gates Foundation.

[CR11] Kane, T. J., & Staiger, D. O. (2012). *Gathering feedback for teaching: Combining high-quality observations with student surveys and achievement gains. Research paper. MET project*. Bill & Melinda Gates Foundation.

[CR12] Kaufman, J. H., & Diliberti, M. K. (2021). *Divergent and inequitable teaching and learning pathways during (and perhaps beyond) the pandemic: Key findings from the American educator panels spring 2021 COVID-19 surveys*. RAND Corporation. Data note: Insights from the American educator panels. Research report. RR-A168-6.

[CR13] Kraft, M. A., & Monti-Nussbaum, M. (2021). The big problem with little interruptions to classroom learning. *AERA Open,**7*, 23328584211028856.

[CR14] Lasagabaster, D., & Sierra, J. M. (2011). Classroom observation: Desirable conditions established by teachers. *European Journal of Teacher Education,**34*(4), 449–463.

[CR15] Lindorff, A., & Sammons, P. (2018). Going beyond structured observations: Looking at classroom practice through a mixed method lens. *ZDM,**50*, 521–534.

[CR16] Marsh, B., & Mitchell, N. (2014). The role of video in teacher professional development. *Teacher Development,**18*(3), 403–417.

[CR17] McLean, L., & Connor, C. M. (2018). *Challenges, benefits, and considerations when conducting classroom video observation research*. SAGE Publications Ltd.

[CR18] National Assessment of Educational Progress. (2022a). *Data informs policy and practice*. Retrieved May 27, 2023, from https://nces.ed.gov/nationsreportcard/about/policy_practice.aspx

[CR19] National Center for Education Statistics, National Assessment of Educational Progress: The Nation’s Report Card. (2022b). Washington, D.C: National Center for Education Statistics, Office of Educational Research and Improvement, US Dept. of Education.

[CR20] Paterson, B. L. (1994). A framework to identify reactivity in qualitative research. *Western Journal of Nursing Research,**16*(3), 301–316. 8036805 10.1177/019394599401600306

[CR21] Postholm, M. B. (2012). Teachers’ professional development: A theoretical review. *Educational Research,**54*(4), 405–429.

[CR22] Pressley, T. (2021). Factors contributing to teacher burnout during COVID-19. *Educational Researcher,**50*(5), 325–327.

[CR23] Walker, M., Worth, J., & Van den Brande, J. (2019). *Teacher workload survey 2019*.

