# Supplementary material for: Expanding Education Researchers’ Access to Classroom Observation Data With a Remote and Cost-Effective Video Data Collection Protocol
Source: Prev Sci. 2024 Mar 22;27(1):6–15. doi: 10.1007/s11121-024-01659-w (PMC12906552; doi:10.1007/s11121-024-01659-w)
Supplement: Supplementary file 1 — Supplementary file1 (DOCX 18 KB) [file 11121_2024_1659_MOESM1_ESM.docx]

**Text to Aid in Guardian Consent Documents**

Dear Parent/Guardian,

My name is <PI NAME>, and I am the Principal Investigator of the <STUDY NAME> at <INSTITUTION>. In this study, we are hoping to <STUDY PURPOSE>.

Your child’s teacher, <INSERT NAME>, is enrolled in our study and recently indicated interest in participating in our video observation portion of the study. During video observations, participating teachers record themselves teaching using our project equipment (remote cameras and secure Internet hotspots). We are asking your child’s teacher to record their instruction during the weeks of <INSERT DATES> this <INSERT SEASON>. During this time, your child’s teacher will record between one and four hours of their instruction over the course of a week. During recording, your child’s classroom, including your child, will be captured using a wide-angle lens placed in a back corner of the classroom. Most of the classroom will be in view at all times, but no one child will be the focus of the observation, and no zoom-in features will be used. Your child’s teacher will be instructed to remove any identifying information (name tags, etc.) from view of the camera, and will be instructed to refrain from using any students’ full name or other identifying information. Any instance of identifying information on the recordings will be permanently trimmed from the video.

Video recordings will be immediately uploaded to secure Google Home accounts managed by project staff, and from there will be downloaded, cleaned, and stored long-term on external hard drives in my secure campus lab. Trained project staff will assess the videos in the lab.

We are asking for your consent to your child being present in the classroom during these video observations. If you consent, your child will be captured on these recordings along with their peers and their teacher. You have the right to decline your child being in view of the camera during these video observations.

Respectfully,

<PI NAME AND SIGNATURE>

**Please make your consent selection below by selecting only one box:**

I consent to my child being present for these video observations.

I DO NOT consent and would like my child to be removed from view of the camera.



Printed Name: ______________________________________

Signature: **_________________________________________**

Date: _____________________________________
