# Supplementary material for: Expanding Education Researchers’ Access to Classroom Observation Data With a Remote and Cost-Effective Video Data Collection Protocol
Source: Prev Sci. 2024 Mar 22;27(1):6–15. doi: 10.1007/s11121-024-01659-w (PMC12906552; doi:10.1007/s11121-024-01659-w)
Supplement: Supplementary file 2 — Supplementary file2 (DOCX 15 KB) [file 11121_2024_1659_MOESM2_ESM.docx]

**Text to Aid in IRB Submissions**

**Description of Protocol:** Those who indicate they are willing to participate in video observations will be contacted by the research team via email and will be asked to provide the school email address of their school principal. Project staff will send an informational letter to each school principal via email describing the purpose and procedures of video observations, potential risks and benefits, data security protocols, and child “opt out” protocols. They will be given an opportunity decline their teacher participating in video observations via email reply to the research team. If a principal declines their teacher participating, no video observations will occur in that teachers’ classroom. All participants with principal approval will be asked to complete an electronic video observation consent form. After completing the consent process, the research team will provide each consented video observation participant with guardian consent letter to send home to the parents/guardians of all students in their class. These letters will inform guardians of all activities, risks and benefits, and data security protocols associated with video observations in their child’s classroom and will ask guardians to consent to or decline their child’s presence during video observations. If a child’s parents decline to their child being present during observations, the teacher will be instructed to either arrange for that child to be out of the classroom for the entire observation, or to move that child out of view of the camera during recording if removing them from the classroom is not feasible.

Each teacher participant who provided consent to participate in video observations will be asked to record observations <#> times from <DATE RANGE>. For each time point, participants will record themselves teaching one to four discreet lessons over the span of one week. Observations will take place using video self-recording equipment provided by project staff. Recording kits will be shipped to participants’ schools and will include a remote Google Nest camera, a secure mobile Internet hot spot, and instructions for conducting the observation. The participant will use these materials to record their lessons, and once finished will ship the kit back using a pre-paid mailing label provided by the research team. Video observations will automatically be uploaded to a secure lab Google Nest account managed by the research team. The research team will check the account daily during active video recording, will remove any new videos that have been uploaded, will check each video for identifiable data and trim accordingly, and will then videos to a secure external hard drive housed in the lab of the PI.

**Video Data Storage and Security:** Teacher participants who complete video self-recordings will be instructed to remove all identifying information of themselves and their students from view of the camera prior to conducting each recording (name tags, etc.). Participants will also be instructed to refrain from using students’ full names or any other identifying information during recorded lessons. Participants will be provided with a laminated sign to post on their classroom door during each recording asking outside parties not to enter. Prior to video assessment and storage, videos will be checked by project investigators for identifiable instances (for example, a student’s full name is used, a name tag displayed on a desk), and these instances will be trimmed from the video. Videos will be stored securely on devices not connected to the Internet in in the on-campus lab of the PI. All assessment of videos will take place in the on-campus lab of the PI on computers equipped with privacy screens which are not visible to any outside parties. Only IRB-approved project members will assess video data. De-identified video data will be stored for a period of 5 years, at which point keyed data will be transferred to long-term data storage service and video data will be permanently destroyed.
