# Supplementary material for: Expanding Education Researchers’ Access to Classroom Observation Data With a Remote and Cost-Effective Video Data Collection Protocol
Source: Prev Sci. 2024 Mar 22;27(1):6–15. doi: 10.1007/s11121-024-01659-w (PMC12906552; doi:10.1007/s11121-024-01659-w)
Supplement: Supplementary file 3 — Supplementary file3 (DOCX 33 KB) [file 11121_2024_1659_MOESM3_ESM.docx]

**Text to Aid in Teacher Participant Recording Instructions**

We are excited to be working with you on the video observation portion of <STUDY NAME>! Please set aside about 20 minutes of time to carefully review these instructions and test your recording equipment prior to conducting your first recording. We recommend doing this during a time when students are not in the room with you (before or after class or during your planning time).

If you would like to schedule a Zoom meeting in the next few days with one of our video equipment experts so that we can go through these steps with you, please email <CONTACT>.

**What you’re doing…**

- We would like you to record yourself teaching lessons to your students. Each lesson should have a clear topic and a beginning, middle, and end. Please do not record students solely completing independent work or taking tests, although it is alright for these activities to be included within a larger lesson (for example, an independent workstation during center rotations).
- Our goal is to capture your typical instruction, and so you can choose which topics you would like to teach and how long each lesson will be. Just do what you would normally do in your class! Most lessons we see last between 40 and 60 minutes, but this is not a strict rule by any means – yours can be shorter, longer, or about the same.
- You will have the camera kit for <TIME SPAN>. We are hoping to collect recordings of <#> lessons from you in that time. You will be paid <$> for each recording we receive.

**What you’ll need…**

- Camera kit (bag containing Google Nest camera and Verizon mobile hotspot). **Please use caution when opening the package and save the box**. You will need to re-use the box to ship the kit back. The camera kit includes…
  - Google Nest camera – box includes wired camera, wall outlet adaptor, blue instruction pamphlet, labels inside box and on instruction pamphlet indicating the camera name (e.g., Nest Cam 1, Nest Cam 2, etc.)
  - Verizon Jetpack mobile hotspot – box includes Jetpack device, instruction pamphlet, power cord, and wall outlet adaptor
  - “Recording in Progress” sign
  - UPS prepaid return shipping label
- A location in your classroom where the camera can be placed to record lessons (see below for details on choosing a location)

**Using the Camera and Hotspot**

1. Open the Verizon Jetpack Mobile Hotspot box and remove the device. Press and hold the power button
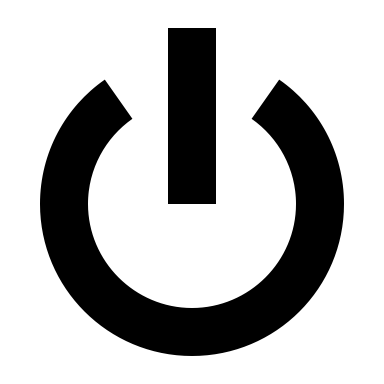
 on the top of the device to turn it on.
   1. Plug the device into the outlet near where you have chosen to place the camera. The battery should be charged, but we ask that you please keep the Jetpack device plugged in at all times during recording to prevent any possible disruption of the connection.
2. Open the Nest Cam box and take out the camera. Plug the camera cord into the wall outlet adaptor and plug into the wall outlet (or if plug into a compatible USB power source without the adaptor) near where you have chosen to place the camera.
3. Your camera will automatically start recording audio and video of your classroom (once the white light on the camera turns green, which should only take a moment, the camera is recording).
4. **The camera MUST be unplugged in between lessons**. Otherwise, it will record anytime there is movement in the classroom, and we will not be able to determine what you intended to record as a “lesson.”

**Placing the Camera and Recording the Lesson**

1. Place the camera in your chosen spot. Your spot should be:
   1. Along the wall of the classroom, away from most foot traffic
   2. Near an electrical outlet with two available plug-ins
   3. On a high surface such as on top of a filing cabinet or shelf (on top of a desk or countertop is also fine if you don’t have a filing cabinet or shelf available)
2. Position the camera so that it is facing the portion of the classroom where you will be conducting most of your lesson. The camera captures a wide-angle view of the classroom, so if it is pointed in the general direction of where your instruction will be taking place, that will be sufficient. Typically, it works best to place the camera on a high surface in the back of the room facing forward to the front of the room.
3. If you have any students whose guardians have requested they be out of view of the camera, move them to an area behind the camera, or to another pre-determined school location.
4. If you would like a research team member to check the live feed of your camera to ensure adequate placement, you can call <CONTACT>.
5. The green light indicating active recording should remain on during the duration of the lesson. If the green light goes out while you are recording, try the troubleshooting steps provided at the bottom of this document.
6. When you have finished the lesson and are ready to stop recording, unplug the camera from the electrical outlet. The recording will end and the video will automatically be uploaded to our Google Home cloud account where we will retrieve it. **PLEASE NOTE:** **You must unplug your camera between lessons, otherwise the camera will record anytime there is motion in the classroom, and we will not be able to determine which section of the video is the “lesson” you intended to record.**
7. Power off the Jetpack device by pressing and holding the power button
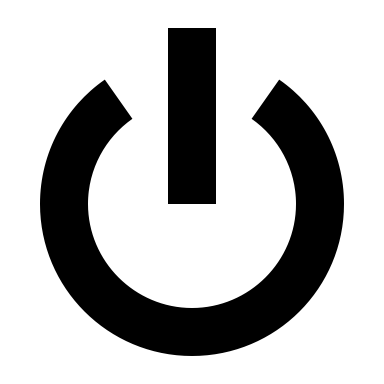
 on the top of the device, then selecting “Shut down,” and “Ok.”
8. Pack the Nest Cam into the Nest Cam box and the Jetpack into the Verizon Jetpack box and store in a safe, out-of-view area in your classroom until you are ready to record your next lesson.
9. Repeat these instructions for each lesson you record throughout your two-week recording period. In between recordings, please store all camera kit equipment safely in your classroom.

**Before/During Recorded Lessons**

1. Before you begin recording each lesson, scan your classroom to see if there is anything in plain view that might identify your students. For example, student name tags with their full name displayed; student art on the wall with their full name displayed. Remove anything that might identify a student.
2. Before you begin recording, place the “observation in progress” sign provided with your equipment on the outside of your classroom door.
3. As you are teaching your recorded lessons, please refrain from using any students’ full (first and last) name. It is fine to use students’ first names.

**Returning the camera kit**

1. Make sure that all the materials are repackaged in the appropriate boxes and then return the boxes to the camera kit bag. Refer to the list on pg. 1 to double check everything is in the bag, being especially aware of chargers and power cords.
2. Place the camera kit bag in the box in which it arrived.
3. Remove the old shipping label.
4. Attach the prepaid return shipping label on the box.
5. Drop off the package at your local UPS store.

**Troubleshooting**

1. If any of the above step-by-step instructions are not working as expected, please email <CONTACT> and we will set up a Zoom call to troubleshoot with you.
2. If the green light on the Google Nest camera goes out during an active recording session:

- Make sure Verizon Jetpack hotspot is connected to the network (will show active “bars” in the top left corner where it says 4G LTE, will show Wi-Fi – ON in lower left)
- Make sure device with Google Home app and camera are both connected to the mobile hotspot Wi-Fi (on the Jetpack device, in the center of the bottom row where it says “Devices” it should show 2 devices connected).
- If the Verizon Jetpack hotspot is connected to the network, and the device you are using to access the Google Home app is connected to the hotspot Wi-Fi, but your camera shows that it is “offline” please try unplugging the camera for a few moments, plugging it back in, and then attempting to connect to the video feed in the Google Home app.
- If the hotspot is connected to the network, and the Google Home app and camera are connected to the Verizon hotspot Wi-Fi network, but you are still having trouble, please reach out via email to <CONTACT>.
