# Supplementary material for: Expanding Education Researchers’ Access to Classroom Observation Data With a Remote and Cost-Effective Video Data Collection Protocol
Source: Prev Sci. 2024 Mar 22;27(1):6–15. doi: 10.1007/s11121-024-01659-w (PMC12906552; doi:10.1007/s11121-024-01659-w)
Supplement: Supplementary file 4 — Supplementary file4 (DOCX 20 KB) [file 11121_2024_1659_MOESM4_ESM.docx]

**Text to Aid in Teacher Consent Documents**

You are being invited to participate in the video observation component of <STUDY NAME>. This consent form tells you about these study activities including their purposes, what you will be asked to do if you decide to take part, and the potential risks and benefits posed to you. Please read the information below before you decide whether or not to participate in these activities.

**Study Purpose:** The purpose of these activities is to learn more about how teachers’ instructional practices influence classroom processes and student outcomes.

**Who is being asked to participate?** You are one of approximately <#> participants invited to participate in these study activities. You are being asked to participate in these study activities because you are <INCLUSION CRITERIA>.

**What will you be asked to do?** If you consent, you will complete self-recordings using project video equipment of your classroom instruction <#> times between <TIME SPAN>. If you do not wish to complete these study activities, there are no known alternatives available to you.

**What are the possible risks and discomforts?** The research team does not expect your participation in this study will expose you to any risks different from those you would normally encounter in daily life. You may experience mild discomfort from being videotaped as you instruct your class.

**What are the potential benefits?** You will not benefit directly from taking part in this research. However the knowledge gained from this study may contribute to our understanding of how teachers’ instructional practices contribute to their students’ classroom experiences and eventual outcomes.

**New findings that could affect your participation:** During the course of this study, we may learn new important information. This may include information that could cause you to change your mind about participating in the study. If any new important information becomes available while you are a participant we will let you know.

**Confidentiality:** Your study data will be handled as confidentially as possible. If results of this study are published or presented, individual names and other personally identifiable information will not be used. All study data will be analyzed and reported in aggregate form, meaning no one person’s data will be assessed or reported individually. To minimize the risks to confidentiality, we will ask for you to remove all identifying information on you or your students from view of the cameras during video recordings. We will also ask that you refrain from using yours’ or your students’ full names during video recordings. Prior to assessing video data, project members will review your recordings and remove any instances where yours or your students’ names or other identifying information is seen or spoken. Lastly, data collected in this study will not be shared outside the research team. We will keep your study data confidential and only those with permission in the research team will have access to information that identifies you. We may have to report certain information for legal or ethical reasons, such as child abuse, or intent to hurt yourself or others. If required, your records may be inspected by authorized personnel in the following groups and agencies: the University of Delaware Institutional Review Board, and the National Science Foundation.

**Use of data collected in future research:** Identifiers about you will be removed from all data, and after such removal data could be used for future research studies without additional informed consent from you or your legally authorized representative.

**Costs and compensation:** There are no costs associated with participating in the study. Any participant who completes video observations will receive <$> per observation.

**Do you have to take part in this study?** Whether or not to take part in these study activities is your decision. If you choose to take part, you have the right to stop at any time with no penalty. If you decide later not to participate, there will be no penalty or loss of benefits to which you are otherwise entitled.

Your decision to stop participation, or not to participate, will not influence current or future relationships with the <RELEVANT ENTITIES>.

If at any time you decide to end your participation on this research study please inform our research team by emailing <CONTACT>. If you stop your participation in the study, we will keep any data collected of you until that point. You will receive compensation for all completed surveys up to the point of your study exit, and then will not receive any additional compensation after you end your participation.

**Institutional Review Board:** This study has been reviewed and approved by the <INSTITUTION> Institutional Review Board, which is a committee formally designated to approve, monitor, and review biomedical and behavioral research involving humans. If you have any questions or concerns about your rights as a research participant, you may contact the <IRB CONTACT>.

**Contact Information.** If you have any questions about the purpose, procedures, or any other issues related to this research study you may contact the Principal Investigator <PI NAME> at <CONTACT>.

**PLEASE MAKE YOUR CONSENT DECISION HERE:**

I have read and understood the information in this form and I agree to participate in the study. I am 18 years of age or older. I have been given the opportunity to ask any questions and those questions have been answered to my satisfaction. I understand that I will be given a copy of this form for my records.

__ I consent to participate in the video observation activities described above

Printed Name: ____________________________________

Signature: ________________________________________

Preferred email address: ___________________________________

Preferred mailing address: __________________________________

Preferred telephone number: _______________________
